# ERK5 Interacts with Mitochondrial Glutaminase and Regulates Its Expression

**DOI:** 10.3390/ijms25063273

**Published:** 2024-03-14

**Authors:** Yolanda María Guillén-Pérez, María Jesús Ortiz-Ruiz, Javier Márquez, Atanasio Pandiella, Azucena Esparís-Ogando

**Affiliations:** 1 Instituto de Investigación Biomédica de Salamanca (IBSAL), 37007 Salamanca, Spain; u162364@usal.es (Y.M.G.-P.); atanasio@usal.es (A.P.); 2 Instituto de Biología Molecular y Celular del Cáncer (IBMCC)-Consejo Superior de Investigaciones Científicas (CSIC), 37007 Salamanca, Spain; 3 Canceromics Laboratory, Departamento de Biología Molecular y Bioquímica, Universidad de Málaga, 29010 Málaga, Spain; marquez@uma.es; 4 Instituto de Investigación Biomédica de Málaga (IBIMA-Plataforma BIONAND), Universidad de Málaga, 29010 Málaga, Spain; 5 Centro de Investigación Biomédica en Red de Cáncer (CIBERONC), 37007 Salamanca, Spain

**Keywords:** proteomics, ERK5, mitochondria, energy metabolism, glutaminase, GLS isoforms, cancer therapy

## Abstract

Many of the biological processes of the cell, from its structure to signal transduction, involve protein–protein interactions. On this basis, our aim was to identify cellular proteins that interact with ERK5, a serine/threonine protein kinase with a key role in tumor genesis and progression and a promising therapeutic target in many tumor types. Using affinity chromatography, immunoprecipitation, and mass spectrometry techniques, we unveiled an interaction between ERK5 and the mitochondrial glutaminase GLS in pancreatic tumor cells. Subsequent co-immunoprecipitation and immunofluorescence studies supported this interaction in breast and lung tumor cells as well. Genetic approaches using RNA interference techniques and CRISPR/Cas9 technology demonstrated that the loss of ERK5 function led to increased protein levels of GLS isoforms (KGA/GAC) and a concomitant increase in their activity in tumor cells. It is well known that the tumor cell reprograms its intermediary metabolism to meet its increased metabolic needs. In this sense, mitochondrial GLS is involved in the first step of glutamine catabolism, one of the main energy sources in the context of cancer. Our data suggest that ERK5 contributes to the regulation of tumor cell energy metabolism via glutaminolysis.

## 1. Introduction

Extracellular-Signal-Regulated Kinase 5 (ERK5) is part of a mitogen-activated protein kinase (MAPK) signaling cascade which includes the kinases MEKK2/3, MEK5, and ERK5 itself. ERK5 contains an N-terminal region with a kinase domain and a carboxy-terminal extension unique within the MAPK family [1]. This C-terminal extension includes a transcriptional activation domain, two proline-rich regions, and nuclear import and export signals [2,3,4]. Agents such as polypeptide growth factors or cellular stress [5,6] activate the upstream kinases MEKK2/3, which trigger the phosphorylation of MEK5 [7]. Once activated, MEK5 phosphorylates ERK5 at the TEY motif within the activation loop, and this causes the activation of ERK5 [8], which triggers the autophosphorylation of multiple sites within its C-terminal tail in addition to the phosphorylation of downstream intermediates [9]. Ultimately, this sequential activation results in cell biological responses such as proliferation [6,10].

Depending on the cell type, the cellular distribution of ERK5 under basal conditions varies from a diffuse cellular pattern to others in which ERK5 is localized in certain cellular compartments [4]. Moreover, it has been reported that the activation of ERK5 may cause the translocation of the protein from the cytosol to the nucleus [11,12,13].

In cancer, the increased expression or activation of MEK5 or ERK5 has been linked to the genesis or progression of several types of tumors [14,15,16,17,18]. Focusing on cell energy metabolism, in recent years, some studies have determined that ERK5 participates in mitochondrial function (reviewed in [14,15]). For instance, the specific loss of ERK5 expression in mouse cardiomyocytes leads to reduced cardiac contractility and mitochondrial dysfunction with repressed fuel oxidation and oxidative damage after consuming a high-fat diet [19].

The non-essential amino acid L-glutamine (Gln) becomes conditionally essential in many pathological states and diseases [20]. Many cancer types, including triple-negative breast cancer and pancreatic and lung cancers, have been shown to be highly dependent on an increased Gln supply to sustain their characteristic uncontrolled growth and proliferation [21]. In fact, the term Gln addiction was coined to describe the strong need for this amino acid as an essential substrate for energy and biosynthetic purposes in cancer cells [22]. The first step in Gln catabolism, referred to as glutaminolysis, involves its deamidation, yielding glutamate and ammonia under the control of the enzyme glutaminase. In mammals, two paralogous genes, located in different chromosomes, encode four different glutaminase isoenzymes [23,24]. The human *GLS* gene, located on chromosome 2, codes the GLS isozymes KGA and GAC, whereas the *GLS2* gene, located on chromosome 12, produces the GLS2 isoenzymes GAB and LGA [25].

Glutaminases play a key role in cancer metabolic reprogramming. Thus, the upregulation of GLS isozymes has been correlated with proliferating stages and malignancy in many types of cancer and experimental tumors [26]. In line with this oncogenic role, c-Myc has been described as an important regulator of Gln metabolism by allowing upregulation of GLS through a miRNA mechanism [27,28,29]. In pancreatic cancer, the transcription factor EB (TFEB) is required to sustain GLS-dependent glutamine catabolism; therefore, it has been proposed as a new potential therapeutic target [30]. Although therapeutic approaches involving the inhibition of glutamine metabolism through GLS in tumors are being developed for clinical trials [31], these formulations need to be combined because monotherapies frequently elicit metabolic stress and resistance mechanisms, regular characteristics of aggressive tumors [32]. 

Combination therapy consisting of the simultaneous targeting of regulatory nodes remains a procedure capable of improving treatment responses [33]. In this sense, the identification of new therapeutic targets and signaling pathways related to glutaminolysis is crucial to allow for the blocking of several related pathways in order to synergistically arrest cancer development.

In the present work, we show that ERK5 interacts with the mitochondrial enzyme glutaminase-GLS (KGA/GAC). Furthermore, this study reveals that ERK5 can regulate glutaminase levels. These results provide a basis for future research to characterize the type of interaction and its implications in the development of future therapeutic strategies.

## 2. Results

### 2.1. Proteomic Studies Identify KGA/GAC as Potential Interactors with ERK5

**Figure 1:**
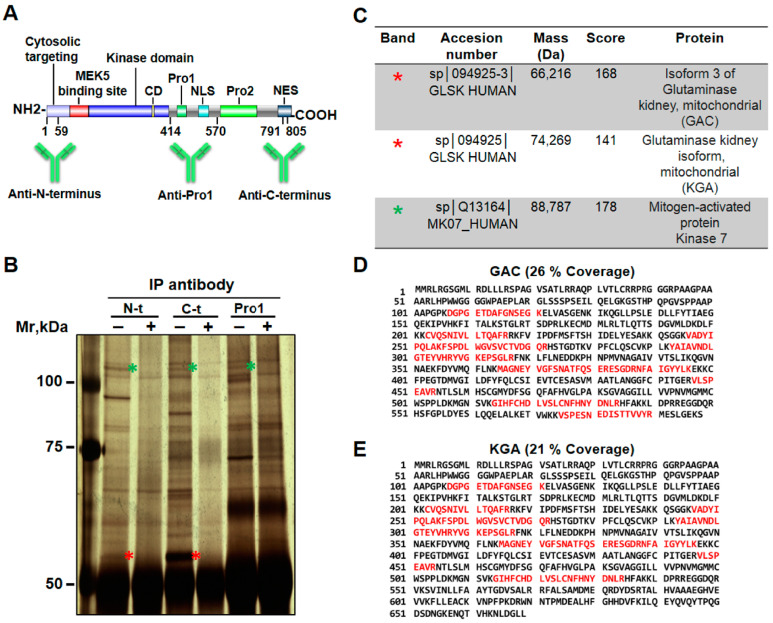
**Proteomic studies: immunoprecipitation.** (**A**) Schematic representation of ERK5, showing the regions recognized by antibodies. (**B**) Whole cell extracts from NP9 cells were immunoprecipitated with the antibodies indicated in (**A**) in the absence or presence of an excess of the fusion protein or peptide against which the antibodies were generated. SDS-PAGE (5–10% gradient gel) was conducted with the samples, and protein bands were visualized by silver staining. Bands highlighted with red and green asterisks were cut out and analyzed using MALDI-TOF MS. (**C**) Data obtained by mass spectrometry (from the bands marked with red and green asterisk) were analyzed using the Mascot search engine against the Swiss-Prot database. Individual scores higher than 59 were considered significant (*p* < 0.05). (**D**,**E**) Matching peptides in the GAC and KGA sequences are highlighted in red, and % coverage values are indicated.

NP9 pancreatic cancer cells were lysed in a mild lysis buffer, and cell lysates were immunoprecipitated with antibodies directed against different domains of ERK5 (the N-terminus, C-terminus, and Pro1 (an internal domain that includes the first proline-rich region and the NLS), see Figure 1A). To identify specific bands interacting with ERK5, these immunoprecipitations were carried out in the presence or absence of antigens used for the generation of three different antisera: fusion proteins in the case of the N-terminus and Pro1 antibodies or a 15-mer C-terminus peptide in the case of the anti-ERK5 C-terminus antibody. The immunoprecipitates were then separated by SDS-PAGE, and the gels were silver-stained (Figure 1B). An inspection of these gels showed protein bands that disappeared when the immunoprecipitations were carried out in the presence of the competing fusion protein or peptide. 

We focused on the bands marked with red and green asterisks (Figure 1B), the latter because they appeared to have a molecular weight compatible with that of ERK5 during the SDS-PAGE procedure and could therefore be used as positive controls for the conditions of the experiment. Mass spectrometry was performed for these bands (Supplementary Figure S1A,C), and the resulting data were analyzed using the Mascot Peptide Mass Fingerprinting search engine, selecting the public database of protein sequences Swiss-Prot. A statistically significant score greater than 59 (*p* < 0.05) was established. From this analysis, the protein identified to have the highest score, in the red asterisk band, matched the mitochondrial protein glutaminase (GLS) in its two isoforms, GAC and KGA, with scores of 168 and 141, respectively (Figure 1C and Supplementary Figure S1B), and sequence coverage values of 26% and 21%, respectively (Figure 1D,E). Moreover, both isoforms were the only hits found above the identity threshold established. An in-depth analysis using mass measurement accuracy in a progressive range between 100 ppm and 10 ppm (Supplementary Table S1) confirmed these isoforms to be the only statistically significant matches. This red asterisk band was also searched for the peptide masses of the *GLS2* gene proteins (LGB and GAB isoforms), but no peptide masses for those proteins were found. Immunoprecipitates corresponding to the anti-ERK5 Pro1 antibody could not be analyzed since the immunoglobulin band covered the red asterisk area in the SDS-PAGE gel (Figure 1B). As anticipated above, the peptide spectrum (Supplementary Figure S1C) corresponding to the band marked with a green asterisk matched the ERK5 protein sequence (Figure 1C and Supplementary Figure S1D). The fact that two antibodies directed against two very different epitopes in the ERK5 sequence yielded the same results strongly suggested the existence of an interaction between ERK5 and GLS.

Affinity chromatography experiments using the anti-ERK5 C-terminus antibody coupled to CNBr-activated Sepharose were performed to confirm the interaction of ERK5 and GLS. The proteins that had been retained on the column due to an affinity interaction with the ERK5 antibody were eluted, resolved on SDS-PAGE gel, and visualized by silver staining (Supplementary Figure S2A). A band migrating with a similar molecular weight to that of GLS (red asterisk) was analyzed. The peptide spectrum (Supplementary Figure S3A) and a proteomic analysis of the band identified the human glutaminase mitochondrial protein (GLS) isoforms GAC and KGA with the highest scores of 225 and 168, respectively (Supplementary Figures S2B and S3B). The sequence coverage values for GAC and KGA were 39% and 29%, respectively (Supplementary Figure S2C,D). Again, as in the immunoprecipitation experiments, both isoforms were the only hits detected with a score well above the value established to reach statistical significance over the entire range of mass tolerance from 100 ppm to 20 ppm (Supplementary Table S2). These results confirmed the data obtained via the previous procedure. 

Taken together, these proteomic data indicate a potential interaction between ERK5 and GLS. Even though the GAC isoform had the highest score, the KGA isoform cannot be excluded because data provided by MALDI-TOF MS are mass measures and not sequencing data. Consequently, both isoforms might interact with ERK5.

### 2.2. Cell Location of ERK5 in Mitochondria

**Figure 2:**
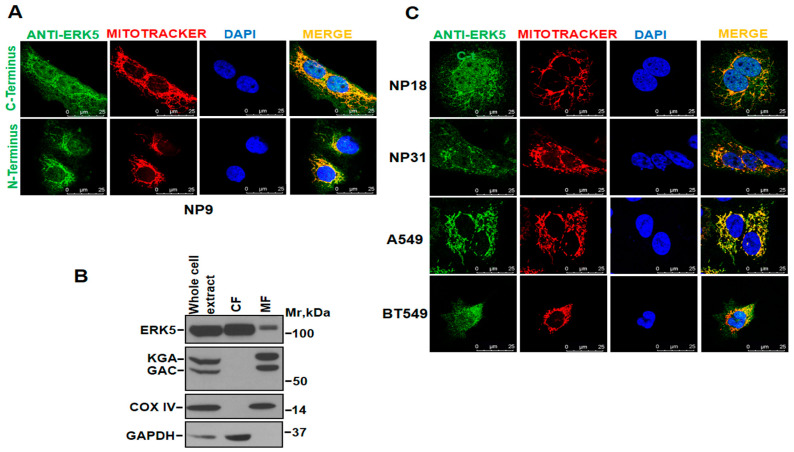
**Immunofluorescence and subcellular fractionation.** (**A**) NP9 cells were incubated with 75 nM of MitoTracker (red) for 30 min at 37 °C in culture to mark the mitochondria. The cells were then fixed and stained with anti-C-terminus or anti-N-terminus ERK5 antibodies, followed by a Cy2-labeled secondary antibody (green). Dapi (blue) was used for staining nuclei. The images were captured with a confocal microscope. Scale bar, 25 µm. Experiments were replicated at least three times. (**B**) Subcellular fractionation was performed on NP9 cells. First, an amount of 80 µg of whole cell extracts (as a control) and 50 µL of either a cytoplasmic fraction (CF) or a mitochondrial fraction (MF) were resolved using SDS-PAGE and analyzed by Western blotting. ERK5 was detected by using the C-terminus antibody. KGA and GAC were detected using the anti-KGA/GAC rabbit antibody. COX IV and GAPDH were used as markers of mitochondrial and cytosolic fractions, respectively. (**C**) NP18, NP31, A549, and BT549 cells were labeled with MitoTracker (red) in a culture and then fixed and stained with the anti-ERK5 C-terminus antibody, followed by a Cy2-labeled secondary antibody (green). Cell nuclei were stained with Dapi (blue). Images were taken with a confocal microscope. Scale bar, 25 µm. Experiments were replicated at least three times.

The discovery of the interaction of ERK5 with the mitochondrial protein glutaminase led us to study whether ERK5 could be in mitochondria. To that end, we carried out immunofluorescence and cell fractionation studies, initially using the NP9 cell line as a model. Immunofluorescence was performed with anti-N-terminus and anti-C-terminus ERK5 antibodies and MitoTracker (used as mitochondrial marker). As shown in Figure 2A, both anti-ERK5 antibodies showed similar patterns of staining. The MitoTracker staining showed that mitochondria were mostly located in the perinuclear regions, conforming to a reticular pattern. Some other stained mitochondria appeared distant from the perinuclear region in a more dot-/rod-like pattern (Figure 2A and Supplementary Figure S4). Overlapping the ERK5 and mitochondrial marker images showed a significant colocalization of the two staining patterns (Figure 2A and Supplementary Figure S4). The above results, obtained with two antibodies targeting different domains of the ERK5 protein, indicate that ERK5 localizes to the mitochondria. 

Cell fractionation studies distinguishing the cytoplasmic fraction from the mitochondria-enriched fraction allowed us to biochemically analyze the subcellular distribution of ERK5 in each fraction. As shown in Figure 2B, Western blot analyses detected an ERK5 signal in the mitochondria-enriched fraction, also accompanied by the presence of KGA/GAC in this organelle. 

Due to the above-demonstrated ERK5-GLS interaction, we then analyzed by immunofluorescence the presence of ERK5 in mitochondria in a wide panel of human cell lines of different tumor types. Among them, human pancreatic (NP18 and NP31), lung (A549) and breast (BT549) cancer cell lines exhibited a mitochondrial ERK5 staining pattern (Figure 2C). Taken together, the above data point to the presence of ERK5 in mitochondria in several tumor cell lines.

### 2.3. ERK5 Coprecipitates and Colocalizes with KGA/GAC

**Figure 3:**
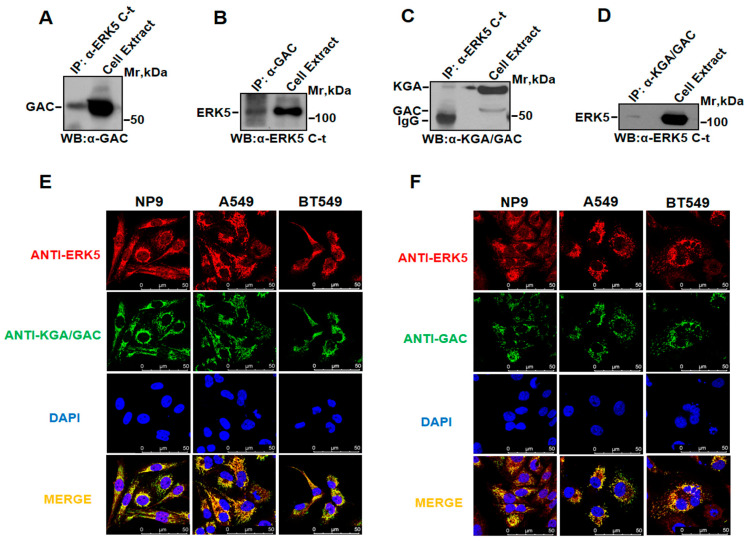
**The co-immunoprecipitation of ERK5 and KGA/GAC and the colocalization of ERK5 and KGA/GAC in situ by immunofluorescence**. (**A**) NP9 cell extracts were immunoprecipitated with the anti-ERK5 antibody, and immunocomplexes were assessed using SDS-PAGE and visualized by Western blotting with an anti-GAC antibody. In addition, 50 µg of whole cell extracts was also analyzed. (**B**) The same as in panel A, with the differences that the immunoprecipitation was performed with the anti-GAC antibody and Western blotting was carried out with the anti-ERK5 antibody. (**C**,**D**) Co-immunoprecipitation data between ERK5 and KGA, using the anti-KGA/GAC rabbit antibody. Additionally, a 50 µg amount of whole cell extracts was also analyzed. (**E**) Representative confocal microscopy images using anti-ERK5 C-terminus (rabbit) and anti-KGA/GAC (mouse) primary antibodies. Cy3-conjugated anti-rabbit (red) and Alexa Fluor^®^ 488-conjugated anti-mouse (green) antibodies were used as secondary antibodies. Nuclei were stained with Dapi (blue). Scale bar, 50 µm. (**F**) Representative confocal microscopy images using anti-ERK5 C-terminus (mouse) and anti-GAC (rabbit) primary antibodies. Alexa Fluor^®^ 568-conjugated anti-mouse (red) and Cy2-conjugated anti-rabbit (green) antibodies were used as secondary antibodies. Nuclei were visualized using Dapi (blue). Scale bar, 50 µm. Immunofluorescence experiments were replicated at least three times.

Proteomic, immunofluorescence, and cell fractionation studies demonstrated the potential existence of an association between ERK5 and KGA/GAC. To add more evidence to these results, co-immunoprecipitation (Co-IP) assays were carried out. Whole-cell NP9 lysates were immunoprecipitated with anti-ERK5 or anti-KGA/GAC antibodies, and immunoprecipitated proteins were resolved using SDS-PAGE. ERK5-KGA/GAC complexes were detected by Western blotting. As shown in Figure 3A, the anti-GAC antibody detected a band with the molecular weight of GAC in the immunoprecipitates obtained with the anti-ERK5 antibody. On the other hand, ERK5 was detected in the anti-GAC immunoprecipitates (Figure 3B). Similarly, KGA was detected in the anti-ERK5 immunoprecipitates (Figure 3C), and ERK5 was detected in the anti-KGA/GAC immunoprecipitates (Figure 3D).

After observing that ERK5 colocalized with the mitochondrial marker MitoTracker and coprecipitated with KGA/GAC, we next checked whether ERK5 colocalized in situ with GLS. To that end, immunofluorescence experiments in NP9, A549, and BT549 cell lines with antibodies directed against ERK5, KGA/GAC (Figure 3E), and the specific antibody against GAC (Figure 3F) were carried out. When confocal microscope images were superimposed, a yellow-orange color indicative of colocalization appeared.

### 2.4. The Functional Relevance of the ERK5-GLS Interaction

**Figure 4:**
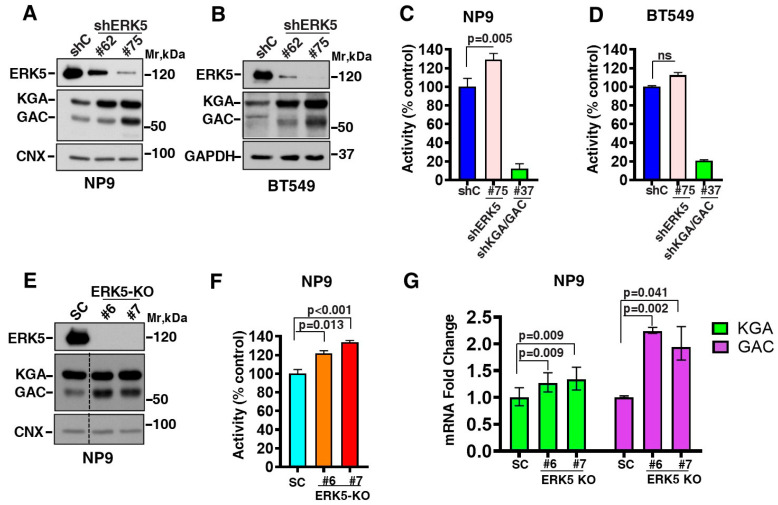
**The effect of ERK5 depletion on GLS expression levels and its enzymatic activity**. The downregulation of ERK5 in the (**A**) NP9 and (**B**) BT549 cell lines was achieved by lentiviral infection with pLKO.1 vectors carrying shRNA sequences for ERK5 (#62 and #75). A nonsense shRNA sequence (shC) was used as a control. An amount of 1 mg of cell extracts was immunoprecipitated with the Pro1 anti-ERK5 antibody, and ERK5 expression was detected by Western blotting with the C-terminus anti-ERK5 antibody. For the detection of KGA/GAC expression levels, 50 µg of NP9 and 100 µg of BT549 cell extracts were used, and Western blots were performed with anti-KGA/GAC rabbit antibody. GAPDH or CNX were used as loading controls. Glutaminase enzymatic activity was determined in (**C**) NP9 and (**D**) BT549 cells with downregulated ERK5. Data are given as the mean ± SD values of three independent experiments with three replicates each. pLKO-shKGA/GAC#37 cells were used as internal controls. (**E**) ERK5 knockout was performed via CRISPR/Cas9 in NP9 cells. SC stands for negative scramble control. ERK5 and KGA/GAC expression and (**F**) glutaminase enzymatic activity were detected in the same way as in the previous panels of this Figure. In all cases, enzyme activity was represented as the % of control (shC for shRNA and SC for the Crispr/Cas9 experiments). *p*-values are indicated. (**G**) Changes in mRNA KGA/GAC levels upon ERK5-KO in NP9 cells (clones #6 and #7) were determined via a qRT-PCR. Data were normalized to *GAPDH* expression and represented as relative quantity (RQ) values of mRNA expression with respect to a control (SC), which was assigned an RQ value of 1. RQ values were calculated according to the 2^−ΔΔCt^ method. Error bars for RQ values were calculated by the equations RQ_min_ = 2^−(ΔΔCt+SD)^ and RQ_max_ = 2^−(ΔΔCt−SD)^. Experiments were repeated twice independently, with three replicates each. *p*-values are indicated. ns: not significant.

To explore the biological consequences of the interaction between ERK5 and KGA/GAC, loss-of-function studies were carried out by genetically manipulating ERK5 levels in two cellular models, pancreatic and breast cancer. Thus, ERK5 expression was downregulated in the NP9 and BT549 cell lines via lentivirus-mediated RNA interference. Two shRNA-ERK5 sequences (#62 and #75) and one noncoding shRNA (shC) sequence were used. The knockdown of ERK5 led to increases in KGA/GAC protein levels in the two cancer cell lines analyzed (Figure 4A,B). Moreover, changes in KGA and GAC expression levels were more notable with the sequence shERK5#75, which was the sequence that achieved the highest ERK5 knockdown. We therefore used this sequence to determine whether this increase in KGA/GAC expression could be translated into an increase in GLS enzymatic activity. As shown in Figure 4C,D, the downregulation of ERK5 was accompanied by an increase in enzymatic activity when compared to a control (shC) in both cell lines. NP9 cells interfered for KGA/GAC expression by shRNA (sequence #37) were used as an internal positive control. As expected, the downregulation of KGA/GAC decreased glutaminase activity.

To reinforce this information, we used CRISPR/Cas9 technology in the NP9 cell line to generate cells lacking ERK5 expression (ERK5-KO). A negative scramble sequence was used as a control (SC). ERK5 expression was efficiently abolished (clones #6 and #7) compared with the control (SC) (Figure 4E). *ERK5* gene disruption showed an increase in glutaminase protein levels which was more evident in the GAC isoform. This fact led to a statistically significant increase in its enzymatic activity compared with the control (SC) (*p* = 0.013 and *p* < 0.001 for clones #6 and #7, respectively; Figure 4F). A quantitative RT-PCR showed higher levels of GLS mRNA (KGA/GAC) in cells lacking ERK5 (clones #6 and #7) compared to control cells (SC) (Figure 4G). Taken together, the genetic depletion of ERK5 led to increased protein levels in GLS and its enzymatic activity. However, the genetic depletion of KGA/GAC did not affect ERK5 expression or activation (measured in terms of pERK5 expression) in NP9, A549, and BT549 cell lines (Supplementary Figure S5A). In a similar way, no changes in ERK5 expression or activation were detected following treatment with the GLS activity inhibitor CB-839 (Supplementary Figure S5B). However, the enzymatic assays showed a decrease in KGA/GAC activity, indicating correct functioning of CB-839 (Supplementary Figure S5B). Therefore, the functional relevance of the ERK5-GLS interaction does not appear to be bidirectional, i.e., ERK5 appears to regulate GLS but not vice versa.

### 2.5. GLS Oligomerization Is Unaffected by ERK5 Downregulation

**Figure 5:**
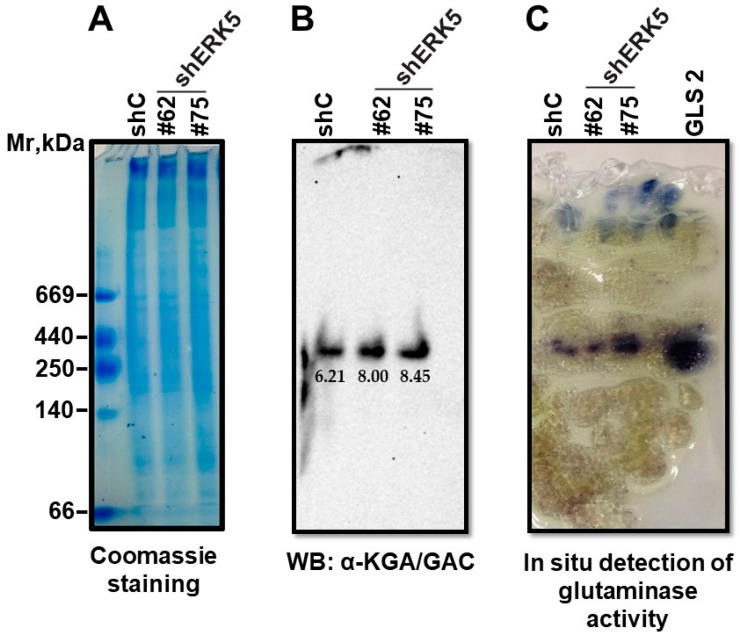
**Native polyacrylamide electrophoresis with CHAPS–agarose gels**. Samples comprising 100 µg of protein extracts from NP9 shC, shERK5#62, and shERK5#75 were loaded on a native gel in a 3–22% gradient. After the procedure, the gel was cut into three parts: (**A**) Coomassie Blue staining indicating molecular mass standards: thyroglobulin (669 kDa), ferritin (440 kDa), catalase (250 kDa), lactate dehydrogenase (140 kDa), and albumin (66 kDa), a mixture for native gels. (**B**) A Western blot for the detection of KGA/GAC with the anti-KGA/GAC mouse antibody. Western blot bands were quantified using Image Studio Lite^TM^ 5.2 analysis software (LI-COR Biosciences), and the values in arbitrary units are shown below each band. (**C**) In situ glutaminase activity was visualized by a double-coupling reaction enzymatic assay using the GDH enzyme and tetrazolium salt. A purified glutaminase protein (GLS2) was used as a positive control.

We wondered whether ERK5 might be involved in the maintenance of the GLS tetramer–monomer balance. To explore this concept, we used native polyacrylamide electrophoresis with CHAPS–agarose gels based on a previously published protocol [34] which also allowed for the analysis of glutaminase activity in situ. Protein extracts from NP9-shC and NP9-shERK5 (sequences #62 and #75) were resolved by electrophoresis under native conditions to preserve the structure and biological activity of GLS. Once the electrophoresis was completed, the gel was divided into three parts (Figure 5). One part was stained with Coomassie Blue (Figure 5A) and used as a guide for molecular masses. The second part was utilized in a Western blot to detect KGA/GAC (Figure 5B). The third part was analyzed to detect in situ glutaminase activity (Figure 5C). Immunodetection with the anti-KGA/GAC antibody (Figure 5B) showed no differences in electrophoretic mobility between NP9-shC and the two sequences of NP9-shERK5. A single band was observed between 440 and 250 kDa of molecular mass, compatible with the molecular mass of the tetramer structure for KGA and/or GAC. Bands of molecular masses corresponding to dimers or monomers were not detected. Therefore, it seems that the genetic downregulation of ERK5 has no influence on the native structure of GLS. 

Regarding the enzymatic activity in situ (Figure 5C), a stained band was detected in lanes corresponding to the NP9-shC and NP9-shERK5 protein extracts, showing as more pronounced in the lane corresponding to shERK5 sequence #75, corroborating the data obtained via the enzyme activity procedure reported previously. A positive control (a recombinant purified GLS2 glutaminase protein) displayed the expected activity, matching the molecular mass of the NP9 lanes.

## 3. Discussion

The ERK5 route regulates several physiological processes, including control over cell duplication or angiogenesis, that are also involved in the generation of an oncogenic phenotype [14,15,35]. While several substrates of this kinase have been defined, knowledge about the participation of the ERK5 route in cellular functions still requires the identification of additional partners. With that purpose, we initiated a study to identify proteins that interact with ERK5, using co-immunoprecipitation (co-IP) as well as affinity chromatography. These studies demonstrated that a protein around 60 kDa of M*_r_* interacted with ERK5. Proteomic analyses of the 60 kDa band showed the presence of peptides corresponding to GLS glutaminase. As mentioned before, two isoforms of GLS, glutaminase C (GAC) and kidney glutaminase (KGA), have been described [23]. Both human glutaminase spliced variants share a high degree of homology [23]. Further evidence supporting the interaction of ERK5 with both the KGA and GAC isoforms was obtained by additional co-IP experiments. Since KGA and GAC are identical at the amino-terminal region and only differ at the C-terminus, it is likely that ERK5 recognizes a sequence or domain similar in both isoforms. Finally, immunofluorescence experiments showed the colocalization of KGA/GAC with ERK5. Together, these studies demonstrated that KGA/GAC are bona fide ERK5-interacting proteins and opened the possibility that the ERK5 signaling pathway could represent a novel route involved in the regulation of glutaminolysis in cancer cells. 

To explore the potential impact of ERK5 on glutaminolysis, loss-of-function genetic experiments were carried out. Both the knockdown of ERK5 using shRNA and the CRISPR/Cas9-mediated knockout of ERK5 resulted in increases in the amounts of KGA and GAC at the protein level. Moreover, the decrease in the levels of ERK5 was accompanied by a significant increase in the transcriptional levels of KGA and GAC, suggesting that ERK5 negatively regulates their expression. While the transcriptional effect of ERK5 on KGA/GAC was not explored further, the fact that the C-terminus tail of ERK5 is endowed with transcriptional activity [9] could explain the direct impact of such a property of ERK5 on the mRNA levels of KGA/GAC. These increases at the mRNA and protein levels also translated into an increase in glutaminase activity. On the other hand, interference with the expression of KGA/GAC did not affect ERK5 expression or phosphorylation status, suggesting that the regulation is not bidirectional. 

The enzymes KGA and GAC show a conformational change from their inactive dimeric form to an active tetrameric form upon binding the activator inorganic phosphate (Pi) [36]. Actually, it is accepted that GLS has a tetrameric structure, based on the pioneering studies of Curthoys and collaborators [37]. In fact, the GLS bands observed in our native gel electrophoresis experiments under the downregulation of ERK5 displayed molecular masses above 250 kDa which are compatible with the structure of a tetramer. On the other hand, the in situ activity staining of GLS in native gels showed a significant enhancement in cells in which ERK5 was downregulated. These findings reinforce the view that ERK5 regulates GLS expression and activity.

Some studies have provided evidence of a relationship between ERK5 and cell metabolism. A recent study demonstrated that in diffuse midline glioma, ERK5 regulates the proglycolytic isoenzyme 6-phosphofructo-2-kinase/fructose-2,6-biphosphatase 3 (PFKFB3) via the activation of transcriptional factor MEF2A [38]. PFKFB3 is involved in the production of fructose-2,6-bisphosphate (F-2,6-BP), a strong activator of 6-phosphofructo-1-kinase (PFK-1), a key regulator of glycolysis. Another recent study found a role of the MEK5-ERK5 axis in the mevalonate pathway in small-cell lung cancer [39]. In leukemia cells, when forced to use oxidative phosphorylation to generate energy, ERK5 expression was increased and accumulated in the mitochondria [40]. ERK5 may function as a mechanism to finely tune GLS levels in tumor cells to assure that the glycolysis pathway is not obliterated by an extremely enhanced glutaminolysis rate. Cancer cell glycolysis impairs antitumor responses via different mechanisms; for example, lactic acid accumulation blocks the activation and migration of T cells and promotes the immune escape of tumor cells [41]. Therefore, rapid GLS ubiquitylation and proteasomal degradation after interacting with ERK5 (probably through the phosphorylation of GLS) might represent a fast route to control both GLS levels and glutaminolysis. 

The relationship between glutaminolysis and signaling routes, in particular with MAPK pathways, has led to a better understanding of cancer metabolic reprogramming. One study in 293T cells associated the Raf-1/MEK2-ERK1/2 MAPK pathway with KGA activation following stimulation with epidermal growth factor (EGF) [42]. Another study identified JNK/c-Jun as a key regulator of GLS. The activation of the transcription factor c-Jun in human breast cancer cells provoked an increase in GLS expression and activity. Furthermore, c-Jun binds to the GLS promoter, increasing its expression [43]. These studies demonstrate the existence of crosstalk between the ERK1/2 and JNK routes and the glutaminolytic pathway.

While the studies reported herein define an interaction of ERK5 with the GLS isoforms KGA and GAC and define the impact of ERK5 on GLS expression levels and activity, the biological consequences of these relationships are yet unclear. In general, ERK5 levels or activity have been linked to an oncogenic phenotype [14]. On the other hand, several studies have shown that GLS is upregulated in different types of cancer [44]. Therefore, the negative regulation of GLS protein levels and activity by ERK5 could be unexpected at first sight; however, more studies are needed in order to understand whether or not this novel interaction between ERK5 and GLS will be another adaptive mechanism of cancer cells to fine-tune and modulate glutaminolysis and glycolysis routes to sustain their growth and proliferation programs.

## 4. Materials and Methods

### 4.1. Reagents and Antibodies

The cell culture media DMEM (41966-029) and RPMI 1640 (21875-034), FBS, penicillin–streptomycin, and trypsin-EDTA were obtained from Gibco™ (Thermo Scientific, Madrid, Spain). A Pierce^TM^ BCA Protein Assay Kit was provided by Thermo Scientific. Immobilon^®^-P PVDF membranes (0.45 μm) were from Millipore Corp. (Darmstadt, Germany). Protein A-Sepharose™ CL-4B, Gamma Bind G-Sepharose™, and CNBr-activated Sepharose™-4B were obtained from GE Healthcare (Uppsala, Sweden). A Silver Stain Plus Kit was obtained from Bio-Rad (Hercules, CA, USA). CB-839 was obtained from SelleckChem (Houston, TX, USA). MitoTracker™ Red CMXRos was obtained from Molecular Probes (Eugene, OR, USA). O-Phthalaldehyde (OPA), L-glutamine, hydrazyne, adenosine diphosphate (ADP), and β-nicotinamide adenine dinucleotide (NAD+) were purchased from Sigma-Aldrich (St. Louis, MO, USA). Glutamate dehydrogenase (GDH) from beef liver (3000 UNITS) was obtained from Roche (Basel, Switzerland). Other generic chemicals were purchased from Sigma-Aldrich, Roche Biochemicals (Mannheim, Germany) or Merck (Darmstadt, Germany). 

The antibodies utilized were anti-Calnexin from Stressgen Bioreagents (Victoria, BC, Canada); anti-Cox IV from Cell Signaling Technologies^®^ (Beverly, MA, USA); and anti-KGA/GAC and anti-GAC from Proteintech^®^ (Manchester, UK). Antibodies against the ERK5 C-terminus, N-terminus, and Pro1 were produced in our laboratory [4,12]. Anti-ERK5 (C7) and anti-GAPDH were obtained from Santa Cruz Biotechnology Inc. (Santa Cruz, CA, USA). Secondary antibodies conjugated to horseradish peroxidase (HRP) were obtained from Bio-Rad Laboratories (Hercules, CA, USA), Jackson Immunoresearch Laboratories, Inc. (West Grove, PA, USA), and GE Healthcare Life Sciences (Piscataway, NJ, USA). Cy3- and Cy2-conjugated antibodies were obtained from Jackson Immunoresearch Laboratories, Inc; Alexa Fluor Plus 488- and Alexa Fluor 568-conjugated antibodies were obtained from Invitrogen (Thermo Scientific). 

### 4.2. Cell Lines and Cell Culture

The human pancreatic tumor cell lines NP9, NP18, and NP31 were established from xenografts orthotopically implanted in nude mice [45] and were generously provided by Dr. Capella (Bellvitge Biomedical Research Institute-IDIBELL, Barcelona, Spain). A549 (CCL-185) and BT549 (HTB-122) cell lines were obtained from the ATCC^®^. Cells were maintained in culture for no longer than 3–4 months and cultured in 5% CO_2_ and 95% air in a humidified atmosphere at 37 °C. Cells were grown in DMEM (NP9, NP31, A549) or RPMI-1640 (NP18, BT549) supplemented with 10% FBS plus antibiotics (penicillin, 100 U/mL, and streptomycin, 100 μg/mL).

### 4.3. Cell Lysis, Immunoprecipitation, and Western Blotting

Cell monolayers were washed twice with PBS and lysed with an ice-cold lysis buffer (20 mM Tris-HCl, pH 7.0, 140 mM NaCl, 50 mM EDTA, 10% glycerol, 1% Nonidet™ P-40, 1 mM PMSF, 1 mM Na_3_O_4_V, 1 µM pepstatin, 1 µg/mL aprotinin, 1 µg/mL leupeptin, 25 mM β-glycerolphosphate, and 10 mM NaF). Then, lysates were centrifuged at 15,000× *g*/10 min/4 °C, and the supernatants (cell extracts) were collected in new tubes. The protein concentration was determined using the BCA assay, according to the manufacturer’s instructions. Immunoprecipitation and Western blotting were performed as described [46,47,48].

### 4.4. Coupling of Anti-ERK5 Antibody to CNBr-Activated Sepharose™ 4B

CNBr-Activated Sepharose™ 4B was selected as a solid matrix to immobilize the antibody and prepare an immunoaffinity column. Briefly, 1 gr of CNBr-Activated Sepharose™ 4B lyophilized powder was suspend in 1 mM HCl and washed for 15 min in the same solution according to the manufacturer’s instructions (GE Healthcare, Little Chalfont, UK). The next step consisted of coupling the ligand to this solid matrix. For that, the column was washed with a coupling buffer (0.1 M NaHCO_3_ at a pH of 8.3, containing 0.5 M NaCl), followed by the incubation of 1.5 mg of the C-terminus anti-ERK5 antibody in the coupling solution. Incubation was performed by rotating the mixture end-over-end overnight at 4 °C. After several washes with the coupling buffer to remove unbound antibody, any remaining active groups in the column were blocked with 0.1 M Tris-HCl at a pH of 8.0 for 2 h at 4 °C. Then, the column was washed with three cycles of alternating pH. Each cycle consisted of a wash with a 0.1 M acetate buffer at a pH of 4.0 containing 0.5 M NaCl, followed by a wash with 0.1 M Tris–HCl at a pH of 8.0 containing 0.5 M NaCl. Finally, the column was washed with phosphate-buffered saline (PBS); for preservation, 0.05% sodium azide was added, and the column was stored at 4 °C until use.

### 4.5. Purification of Proteins Interacting with ERK5

NP9 cell protein extracts (obtained as described in Section 4.3) were immunoprecipitated using antibodies directed against three regions of ERK5: one against the N-terminus, one against the C-terminus, and another against an internal region, including the first proline-rich region and nuclear localization signal (NLS) (anti-ERK5-Pro1). In parallel, peptide (in the case of the C-terminus antibody) or fusion proteins (in the case of the N-terminus or Pro1 antibodies) against which the antibodies were generated were added to the immunoprecipitation. Immunoprecipitates were resolved by SDS-PAGE in a 5% to 10% gradient.

Alternatively, purification of the ERK5-interacting proteins was carried out by immunoaffinity chromatography. NP9 cell protein extracts were incubated with a CNBr-Activated Sepharose™ 4B-anti-ERK5 C-terminus affinity column for 2 h at 4 °C under gently rocking shaking. After incubation, the flow-through was discarded and the column was washed with a buffer (10 mM Tris–-HCl at a pH of 7.5, containing 150 mM NaCl, 2 mM EDTA, 1 mM PMFS, 25 mM β-glycerolphosphate, 10 mM NaF, and 1 mM Na_3_O_4_V) until the absorbance in the flow-through was ≤0.010, measured at 280 nm in a spectrophotometer. Next, protein complexes bound to the solid matrix were eluted by applying to the column 2 mL of 0.1 M glycine at a pH of 2.6 and carrying out incubation for 10 min under agitation. This step was performed three times. The three eluates were neutralized with 1 M Tris–HCl at a pH of 8.0 at a rate of 100 µL for each mL of eluate, pooled, and dialyzed and concentrated in a PBS buffer using an Amicon^®^ Ultra Centrifugal filter 10K (Merck Millipore, Darmstadt, Germany). Concentrated proteins were separated by SDS-PAGE electrophoresis in a 5% to 15% gradient. 

In both strategies, the gels were stained with a silver stain (Silver Stain Plus™ Kit, Bio-Rad, Hercules, CA, USA), and bands of interest were cut out and analyzed by mass spectrometry.

### 4.6. Identification of ERK5-Interacting Proteins by Mass Spectrometry (MALDI-TOF)

In the electrophoretic bands of interest, the digestion of proteins, the mass determination of tryptic peptides, and the identification of protein complexes were performed at the Proteomics Facility of the Centro de Investigación del Cáncer (Salamanca, Spain). The samples were analyzed on an Ultraflex MALDI-TOF mass spectrometer (Bruker-Franzen Analytic GmbH, Bremen, Germany). Raw spectra were processed and analyzed in FlexAnalysis 3.0 (Bruker Daltonics) software. The generated peaks were analyzed using Mascot Server (version 2.3.02, Matrix Science Ltd., London, UK) and searched against the SwissProt database, restricted to *Homo sapiens*, with the following parameters: up to one missed tryptic cleavage, mass accuracy values of 100, 50, 40, 30, 20, and 10 ppm, carbamidomethyl cysteine as fixed modification, and methionine oxidation as variable modification. Mascot scores with a value greater than 59 were considered as significant (*p* < 0.05).

### 4.7. Immunofluorescence Studies

An immunofluorescence procedure was carried out as described [4,12]. For mitochondrial labeling, live cells were incubated in a growth medium with 75 nM MitoTracker™ Red CMXRos for 30 min before the immunofluorescence procedure was performed. Samples were analyzed by confocal immunofluorescence microscopy using a Leica TSC SP5 (Leica Microsystems, Barcelona, Spain) and the associated Leica Application Suite Advanced Fluorescence (LAS AF version 2.7.3.9723; Leica Microsystems) software.

### 4.8. Cellular Fractionation

Cytoplasmic and mitochondrial fractions were prepared following the protocol described by Ceballos et al. [49]. Cells were trypsinized, collected, and centrifuged at 220× *g* for 3 min at 4 °C. The cell pellet was washed 3 times with PBS and then resuspended in a cytoplasmic lysis buffer (250 mM sucrose, 70 mM KCl, 137 mM NaCl, 4.3 mM Na_2_HPO_4_, 1.4 mM KH_2_PO_4_ pH 7.2, and 200 μg/mL digitonin) supplemented with protease and phosphatase inhibitors and incubated on ice to permeabilize the cells (around 5–10 min). Cell membrane permeabilization was checked using Trypan Blue staining, and samples were then centrifuged at 1000× *g* for 5 min at 4 °C. The supernatant was collected as the cytoplasmic fraction. The pellet was resuspended in a volume (equal to the volume of the cytoplasmic fraction) of mitochondrial lysis buffer (50 mM Tris-HCl pH 7.4, 150 mM NaCl, 2 mM EDTA, 2 mM EGTA, 0.2% (*v*/*v*) Triton X-100, and 0.3% NP-40) supplemented with protease and phosphatase inhibitors and incubated for 5 min on ice; it was then centrifuged at 10,000× *g* for 10 min at 4 °C, and the supernatant was collected as the mitochondrial fraction. Both fractions were analyzed using SDS-PAGE and Western blotting.

### 4.9. Lentiviral Infection and RNA Interference CRISPR/Cas9

TCR lentiviral pLKO.1 vectors bearing shRNA sequences for human *ERK5* (TRCN0000010262 and TRCN0000010275), human *KGA/GAC* (TRCN0000051135, TRCN0000051136 and TRCN0000051137), and a nonsense shRNA sequence (control) were used and purchased from Dharmacon (Lafayette, CO, USA). Lentiviral production was performed as described [18], and target cells were infected. Forty-eight hours after infection, positive transduced cells were selected using puromycin at 3 μg/mL (NP9 and A549) or 2.5 μg/mL (BT549) for a minimum of three days. 

Cells lacking human *ERK5* were obtained using a CRISPR/Cas9 knockout kit (reference KN200655) from OriGene (Rockville, MD, USA). The procedure was conducted according to the manufacture’s procedure. 

### 4.10. Glutaminase Activity Assays

GA activity was measured by either the OPA method that detects ammonia formation or by a double-coupling reaction method that measures glutamate production indirectly. GA activity using the quantification of ammonia produced was assayed essentially as described by Heini et al. [50], with some modifications [51]. Briefly, exponentially growing cells were trypsinized and counted. NP9 (15 × 10^6^), A549 (7.5 × 10^6^), and BT549 (30 × 10^6^) cells were washed with PBS and centrifuged at 500× *g*/3 min. The cell pellets were submitted to three freeze–thaw cycles, inducing mechanical breaking of the cell membranes, and then resuspended with 250 µL of extraction buffer (150 mM K_2_HPO_4_; pH: 8.0). Aliquots of this mixture were combined with a reaction buffer, as described before [51]. The absorbance was then measured at 410 nm in a microplate reader (Ultra Evolution Tecan, Mannedorf, Switzerland). 

A GA assay using the double-coupling reaction consists of two reactions. In the first reaction, L-glutamine is hydrolyzed to L-glutamate and ammonium by the phosphate-activated glutaminase (PAG) enzyme. In the second reaction, the enzyme glutamate dehydrogenase (GDH) catalyzes the reaction of glutamate to α-ketoglutarate, producing an equivalent amount of NADH per molecule of glutamate. For this assay, the procedure was carried out as previously described [52] with some modifications. For the first reaction, 20 µg of protein cell extract in a 10 µL volume (obtained as described in Section 2.3) was mixed with 20 µL of reaction buffer (300 mM K_2_HPO_4_, 0.4 mM EDTA, and 100 mM Tris–acetate at a pH of 8.6) and 20 µL of 40 mM glutamine as a substrate and placed in a clear 96-well flat-bottom microplate (Greiner Bio-one, ref. 655101). The mixture was incubated at room temperature for 10 min, and the reaction was then stopped with 5 µL of 2.13 N HCl. Afterwards, 200 µL of the second reaction buffer (80 mM Tris–HCl pH 9.4, 200 mM hydrazine, 0.25 mM ADP, and 1.7 mM NAD+, containing 7.5 U/mL of the GDH enzyme) was added to each well. The GDH enzyme was the last component added to prevent the reaction from starting. The plate was incubated at room temperature for 1 h. The NADH formed was measured at 340 nm in a microplate reader (BioTek Eon™ Spectrophotometer, Winooski, VT, USA). Three blanks were used: the first contained all the reaction components, omitting the sample; the second contained the sample without inorganic phosphate (Pi); and the third contained the sample without glutamine. The blank with Pi omitted guaranteed that we were measuring GA activity without contamination from other glutamine amidotransferases acting independently of Pi, which may affect the results in crude samples. The specific activity was calculated by dividing the enzyme activity by the sample protein concentration. 

### 4.11. RNA Extraction, cDNA Synthesis, and Quantitative Real-Time PCR

Total RNA was isolated from cells using an RNeasy^®^ Mini Kit (Qiagen, Hilden, Germany), following the manufacturer´s instructions. The quantity and purity of RNA were checked using a NanoDrop^®^ ND-1000 spectrophotometer (Thermo Fisher Scientific, Waltham, MA, USA). The reverse-transcription reaction was carried out to obtain cDNA synthesis using M-MLV reverse transcriptase (Invitrogen by Thermo Scientific, Waltham, CA, USA). A real-time qPCR was performed with SYBR Green PCR Supermix (Bio-Rad Laboratories, Madrid, Spain), using the StepOnePlus™ Real-Time PCR system (Applied Biosystems™, Bedford, MA, USA). Primer sequences, designed with the Primer3 software online tool, were as follows: forward—5′-GCTGTGCTCCATTGAAGTGA-3′ and reverse—5′-TCCAATGGTCCAAAGGAATG-3′ for human *GAC*; forward—5′-AGAACAGCACTCCATGTAGCT-3′ and reverse—5′-CCCGTTCTCAGAATCTCCTTGA-3′ for human *KGA*; and forward—5′-TGCACCACCAACTGCTTAGC-3′ and reverse—5′-GGCATGGACTGTGGTCATGAG-3′ for human *GAPDH*. All amplifications were performed in triplicate from two independent experiments. mRNA expression was normalized to *GAPDH*. The relative quantitation of *KGA/GAC* gene expression was performed using the 2^−ΔΔCt^ method [53].

### 4.12. Native Polyacrylamide Gel Electrophoresis In Situ Glutaminase Activity Assay

Native electrophoresis was performed using the CHAPS–agarose system composed of 3–22% acrylamide gradient gels as previously reported [34]. A commercial mixture of the following proteins was used for molecular mass standards: thyroglobulin (669 kDa), ferritin (440 kDa), catalase (250 kDa), lactate dehydrogenase (140 kDa), and albumin (66 kDa). These standards were stained with a Coomassie blue solution for native gels based on the protocol of Dyaballa and Metzger [54].

For Western blotting, the native gels were washed and equilibrated in a transfer buffer (48 mM Tris, 39 mM glycine, 10% methanol, and 0.05% SDS; pH: 9.2) and transferred to PVDF membranes using the Trans-Blot^®^ SD Semi-Dry Electrophoretic Transfer Cell (Bio-Rad).

For the detection of in situ glutaminase activity, the native gels were washed in 90 mM Tris–HCl at a pH of 8.4 at 4 °C, and the procedure was then carried out as described in [34].

### 4.13. Statistical Analyses

Data were statistically analyzed using the SPSS 22.0 software package (SPSS Inc., Chicago, IL, USA). For the enzyme activity assay in cells lacking ERK5 (CRISPR/Cas9), a preliminary verification of the homogeneity of the experiments (reproducibility) was performed using the independent-samples *t*-test. Data were then analyzed using a one-way ANOVA followed by Tukey´s post hoc multiple-comparison test among groups. For the enzyme activity assay, a non-parametric Mann–Whitney U test was performed to evaluate the difference between the shC and shERK5#75 in the NP9 and BT549 cell lines. To check for statistical differences in gene expression between the control (SC) and ERK5-KO in the NP9 cell line, non-parametric Kruskal–Wallis and pairwise post hoc tests were used. In all statistical analysis, *p* < 0.05 was considered to indicate a statistically significant difference. Quantifications in bar graphs are presented as ±SD values. Graphs were generated using GraphPad Prism, version 8 (GraphPad Software, Inc., San Diego, CA, USA). All experiments were repeated at least two times. 

## 5. Conclusions

In the present work, we report a link between the ERK5 route and the glutaminolysis pathway. We show that ERK5 interacts with the GLS isoforms KGA and GAC and colocalizes with these enzymes in the mitochondria. Furthermore, this study reveals that ERK5 can regulate GLS levels. Since the glutaminolytic pathway appears critical in the reprogramming of tumor cell metabolism to satisfy the increased energy needs of these cells, our results create a novel opportunity for modulating the glutaminolytic pathway by acting on the ERK5 route.

## Data Availability

Data are contained within the article and supplementary materials.

## Supplementary Materials

The following supporting information can be downloaded at https://www.mdpi.com/article/10.3390/ijms25063273/s1.
