# Supplementary material for: ERK5 Interacts with Mitochondrial Glutaminase and Regulates Its Expression
Source: Int J Mol Sci. 2024 Mar 14;25(6):3273. doi: 10.3390/ijms25063273 (PMC10970367; doi:10.3390/ijms25063273)
Supplement: Supplementary file 1 [file ijms-25-03273-s001.zip › ijms-2880252-supplementary.pdf]

International Journal of
Molecular Sciences

MDPI

Supplementary Materials

ERK5 Interacts with Mitochondrial Glutaminase and regulates its expression

Yolanda María Guillén-Pérez, María Jesús Ortiz-Ruiz, Javier Márquez, Atanasio Pandiella and Azucena Esparís-Ogando

A

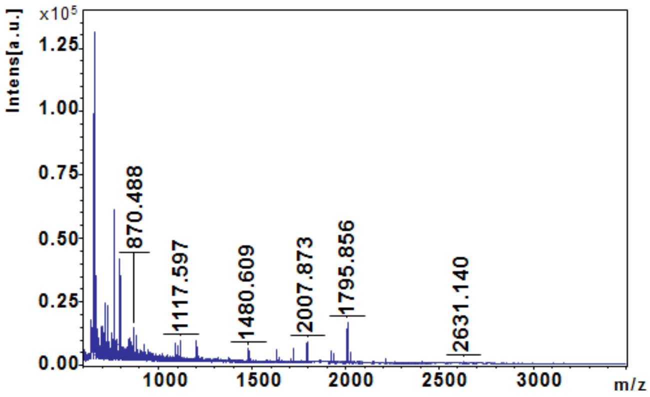

B

1. sp|O94925-3|GLSK_HUMAN **Mass:** 66216 **Score:** 168 **Expect:** 6.7e-013 **Matches:** 13

Isoform 3 of Glutaminase kidney isoform, mitochondrial OS=Homo sapiens OX=9606 GN=GLS

Observed	Mr(expt)	Mr(calc)	ppm	Start End	Miss	Ions	Peptide
870.4881	869.4808	869.4971	-18.66	447 - 454	0		R.VLSPEAVR.N
1088.5519	1087.5446	1087.5702	-23.53	388 - 396	0		R.NFAIGYYLK.E
1105.5841	1104.5768	1104.5927	-14.43	308 - 317	1		R.YVGKEPSGLR.F
1117.5967	1116.5894	1116.6179	-25.53	246 - 255	0		K.VADYIPQLAK.F
1480.6094	1479.6021	1479.6114	-6.28	107 - 121	0		K.DGPGETDAFGNSEGK.E
1632.8045	1631.7973	1631.7943	1.79	383 - 396	1		R.ESGDRNFAIGYYLK.E
1648.8794	1647.8721	1647.8767	-2.77	203 - 216	0		K.CVQSNIVLLTQAFR.R
1720.8520	1719.8448	1719.8580	-7.71	293 - 307	0		K.YAIAVNDLGTEYVHR.Y
1795.8557	1794.8484	1794.8636	-8.45	575 - 590	0		K.VSPESNEDISTTVVYR.M
1922.9147	1921.9074	1921.8993	4.21	256 - 272	0		K.FSPDLWGVSVCTVDGQR.H
2007.8726	2006.8653	2006.8792	-6.93	365 - 382	0		K.MAGNEYVGFSNATFQSER.E
2023.8614	2022.8541	2022.8741	-9.90	365 - 382	0		K.MAGNEYVGFSNATFQSER.E+ Oxidation (M)
2631.1399	2630.1326	2630.1907	-22.08	514 - 534	0		K.GIHFCHDLVSLCNFHNYDNLR.H

No match to: 1202.6034, 1936.9142

2. sp|O94925|GLSK_HUMAN **Mass:** 74269 **Score:** 141 **Expect:** 3.4e-010 **Matches:** 12

Glutaminase kidney isoform, mitochondrial OS=Homo sapiens OX=9606 GN=GLS PE=1 SV=1

Observed	Mr(expt)	Mr(calc)	ppm	Start End	Miss	Ions	Peptide
870.4881	869.4808	869.4971	-18.66	447 - 454	0		R.VLSPEAVR.N
1088.5519	1087.5446	1087.5702	-23.53	388 - 396	0		R.NFAIGYYLK.E
1105.5841	1104.5768	1104.5927	-14.43	308 - 317	1		R.YVGKEPSGLR.F
1117.5967	1116.5894	1116.6179	-25.53	246 - 255	0		K.VADYIPQLAK.F
1480.6094	1479.6021	1479.6114	-6.28	107 - 121	0		K.DGPGETDAFGNSEGK.E
1632.8045	1631.7973	1631.7943	1.79	383 - 396	1		R.ESGDRNFAIGYYLK.E
1648.8794	1647.8721	1647.8767	-2.77	203 - 216	0		K.CVQSNIVLLTQAFR.R
1720.8520	1719.8448	1719.8580	-7.71	293 - 307	0		K.YAIAVNDLGTEYVHR.Y
1922.9147	1921.9074	1921.8993	4.21	256 - 272	0		K.FSPDLWGVSVCTVDGQR.H
2007.8726	2006.8653	2006.8792	-6.93	365 - 382	0		K.MAGNEYVGFSNATFQSER.E
2023.8614	2022.8541	2022.8741	-9.90	365 - 382	0		K.MAGNEYVGFSNATFQSER.E+ Oxidation (M)
2631.1399	2630.1326	2630.1907	-22.08	514 - 534	0		K.GIHFCHDLVSLCNFHNYDNLR.H

No match to: 1202.6034, 1795.8557, 1936.9142

C

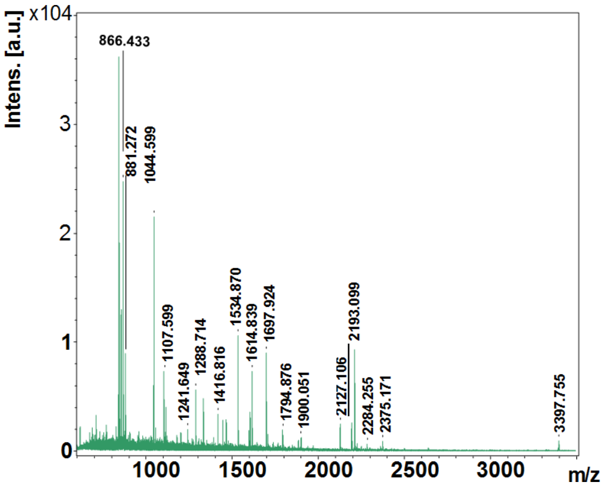

D

Acccesion	Mass	Score	Description
1. sp|Q13164|MK07_HUMAN	88787	178	Mitogen-activated protein kinase 7 OS=Homo sapiens OX=9606 GN=MAPK7 PE=1 SV=2
2. sp|Q13164-3|MK07_HUMAN	59690	158	Isoform 3 of Mitogen-activated protein kinase 7 OS=Homo sapiens OX=9606 GN=MAPK7
3. sp|Q13164-2|MK07_HUMAN	73628	131	Isoform 2 of Mitogen-activated protein kinase 7 OS=Homo sapiens OX=9606 GN=MAPK7
4. sp|Q13164-4|MK07_HUMAN	50690	93	Isoform 4 of Mitogen-activated protein kinase 7 OS=Homo sapiens OX=9606 GN=MAPK7

Supplementary Figure S1. **(A)** MALDI-TOF mass spectrum of the trypsin-digested red asterisk band (peptide mass fingerprint with m/z value).**(B)** Mascot search results for protein identification by peptide mass fingerprint against Swiss Prot database. Comparison of observed and predicted tryptic peptides. **(C)** MALDI-TOF mass spectrum of the trypsin-digested green asterisk band (peptide mass fingerprint with m/z value). **(D)** Mascot search results for protein identification by peptide mass fingerprint against Swiss Prot database.

A

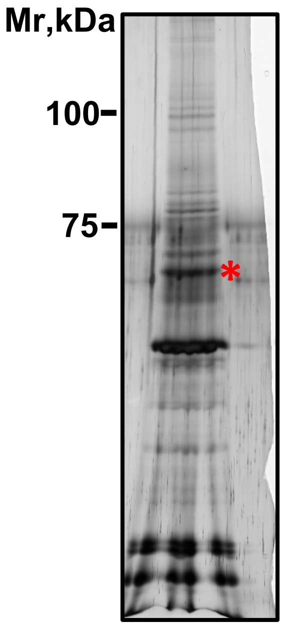

CNBr-α-ERK5 C-t

B

Band	Accesion number	Mass (Da)	Score	Protein
*	sp|094925-3| GLSK HUMAN	66216	225	Isoform 3 of Glutaminase kidney, mitochondrial (GAC)
*	sp|094925| GLSK HUMAN	74269	168	Glutaminase kidney isoform, mitochondrial (KGA)

C

GAC (39 % Coverage)

```
  1  MMRLRGSGML  RDLLLRSPAG  VSATLRRAQP  LVTLCRRPRG  GGRPAAGPAA
 51  AARLHPWWGG  GGWPAEPLAR  GLSSSPSEIL  QELGKGSTHP  QPGVSPPAAP
101  AAPGPKDGPG  ETDAFGNSEG  KELVASGENK  IKQGLLPSLE  DLLFYTIAEG
151  QEKIPVHKFI  TALKSTGLRT  SDPRLKECMD  MLRLTLQTTS  DGVMLDKDLF
201  KKCVQSNIVL  LTQAFRRKFV  IPDFMSFTSH  IDELYESAKK  QSGGKVADYI
251  PQLAKFSPDL  WGVSVCTVDG  QRHSTGDTKV  PFCLQSCVKP  LKYAIAVNDL
301  GTEYVHRYVG  KEPSGLRFNK  LFLNEDDKPH  NPMVNAGAIV  VTSLIKQGVN
351  NAEKFDYVMQ  FLNKMAGNEY  VGFSNATFQS  ERESGDRNFA  IGYYLKEKKC
401  FPEGTDMVGI  LDFYFQLCSI  EVTCESASVM  AATLANGGFC  PITGERVLSP
451  EAVRNTLSLM  HSCGMYDFSG  QFAFHVGLPA  KSGVAGGILL  VVPNVMGMMC
501  WSPPLDKMGN  SVKGIHFCHD  LVSLCNFHNY  DNLRHFAKKL  DPRREGGDQR
551  HSFGPLDYES  LQQELALKET  VWKKVSPESN  EDISTTVVYR  MESLGEKS
```

D

KGA (29 % Coverage)

```
  1  MMRLRGSGML  RDLLLRSPAG  VSATLRRAQP  LVTLCRRPRG  GGRPAAGPAA
 51  AARLHPWWGG  GGWPAEPLAR  GLSSSPSEIL  QELGKGSTHP  QPGVSPPAAP
101  AAPGPKDGPG  ETDAFGNSEG  KELVASGENK  IKQGLLPSLE  DLLFYTIAEG
151  QEKIPVHKFI  TALKSTGLRT  SDPRLKECMD  MLRLTLQTTS  DGVMLDKDLF
201  KKCVQSNIVL  LTQAFRRKFV  IPDFMSFTSH  IDELYESAKK  QSGGKVADYI
251  PQLAKFSPDL  WGVSVCTVDG  QRHSTGDTKV  PFCLQSCVKP  LKYAIAVNDL
301  GTEYVHRYVG  KEPSGLRFNK  LFLNEDDKPH  NPMVNAGAIV  VTSLIKQGVN
351  NAEKFDYVMQ  FLNKMAGNEY  VGFSNATFQS  ERESGDRNFA  IGYYLKEKKC
401  FPEGTDMVGI  LDFYFQLCSI  EVTCESASVM  AATLANGGFC  PITGERVLSP
451  EAVRNTLSLM  HSCGMYDFSG  QFAFHVGLPA  KSGVAGGILL  VVPNVMGMMC
501  WSPPLDKMGN  SVKGIHFCHD  LVSLCNFHNY  DNLRHFAKKL  DPRREGGDQR
551  VKSVINLLFA  AYTGDVSALR  RFALSAMDME  QRDYDSRTAL  HVAAAEGHVE
601  VVKFLLEACK  VNPFPKDRWN  NTPMDEALHF  GHHDVFKILQ  EYQVQYTPQG
651  DSDNGKENQT  VHKNLDGLL
```

Supplementary Figure S2. **Proteomic studies. Affinity chromatography**. **(A)** Whole cell extracts from NP9 cells were subjected to affinity immunochromatography. Proteins eluted from the affinity column were resolved on SDS-PAGE and protein bands were visualized by silver staining. The band highlighted with a red asterisk was cut out and analyzed by MALDI-TOF MS. **(B)** Data obtained by mass spectrometry were analyzed using the Mascot search engine against the Swiss-Prot database. Individual scores higher than 59 were considered significant ($p<0.05$). **(C-D)** Matching peptides in the GAC and KGA sequences are highlighted in red and % coverage is indicated.

A

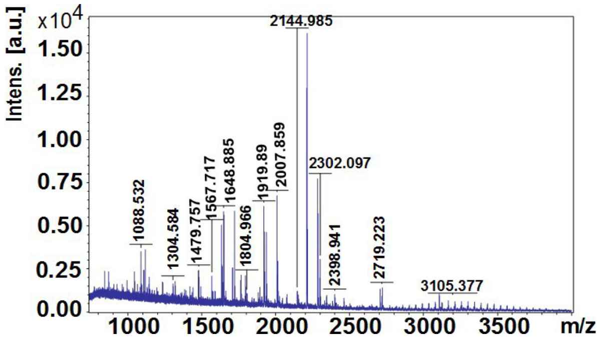

B

1. sp|O94925-3|GLSK_HUMAN **Mass:** 66216 **Score:** 225 **Expect:** 1.3e-018 **Matches:** 20

Isoform 3 of Glutaminase kidney isoform, mitochondrial OS=Homo sapiens OX=9606 GN=GLS

Observed	Mr(expt)	Mr(calc)	ppm	Start End	Miss	Ions	Peptide
1088.5316	1087.5243	1087.5702	-42.19	388 - 396	0	---	R.NFAIGYYLK.E
1105.5586	1104.5513	1104.5927	-37.50	308 - 317	1	---	R.YVGKEPSGLR.F
1117.5779	1116.5706	1116.6179	-42.33	246 - 255	0	---	K.VADYIPQLAK.F
1304.5841	1303.5768	1303.6271	-38.56	355 - 364	0	---	K.FDYVMQFLNK.M
1575.7985	1574.7912	1574.8313	-25.45	280 - 292	0	---	K.VPFCLQSCVKPLK.Y
1632.7829	1631.7756	1631.7943	-11.47	383 - 396	1	---	R.ESGDRNFAIGYYLK.E
1648.8546	1647.8473	1647.8767	-17.80	203 - 216	0	---	K.CVQSNIVLLTQAFR.R
1720.8507	1719.8434	1719.8580	-8.49	293 - 307	0	---	K.YAIAVNDLGTEYVHR.Y
1795.8632	1794.8559	1794.8636	-4.26	575 - 590	0	---	K.VSPESNEDISTTVVYR.M
1804.9655	1803.9582	1803.9778	-10.83	203 - 217	1	---	K.CVQSNIVLLTQAFRR.K
1920.9215	1919.9142	1919.9854	-37.05	86 - 106	0	---	K.GSTHPQPGVSPPAAPAAPGPK.D
1922.8947	1921.8874	1921.8993	-6.18	256 - 272	0	---	K.FSPDLWGVSVCTVDGQR.H
1923.9209	1922.9136	1922.9585	-23.35	574 - 590	1	---	K.KVSPESNEDISTTVVYR.M
2007.8592	2006.8519	2006.8792	-13.60	365 - 382	0	---	K.MAGNEYVGFSNATFQSER.E
2024.9591	2023.9518	2024.0500	-48.51	184 - 201	1	---	R.LTLQTTSDGVMLDKDLFK.K
2074.9705	2073.9632	2074.0371	-35.62	551 - 568	0	---	R.HSFGPLDYESLQQELALK.E
2144.9847	2143.9774	2144.0361	-27.35	347 - 364	1	---	K.QGVNNAEKFDYVMQFLNK.M
2302.0969	2301.0896	2301.1610	-31.01	273 - 292	1	---	R.HSTGDTKVPFCLQSCVKPLK.Y
2408.0974	2407.0901	2407.0775	5.23	107 - 130	1	---	K.DGPGETDAFGNSEGKELVASGENK.I
2649.2184	2648.2111	2648.2290	-6.74	256 - 279	1	---	K.FSPDLWGVSVCTVDGQRHSTGDTK.V

No match to: 1131.7571, 1479.7565, 1567.7170, 1763.7793, 1891.8845, 1936.8856, 2013.0567, 2155.1226, 2330.0893, 2384.9964, 2398.9411, 2401.9405, 2461.0789, 2705.0749, 2719.2274

2. sp|O94925|GLSK_HUMAN **Mass:** 74269 **Score:** 168 **Expect:** 6.7e-010 **Matches:** 17

Glutaminase kidney isoform, mitochondrial OS=Homo sapiens OX=9606 GN=GLS PE=1 SV=1

Observed	Mr(expt)	Mr(calc)	ppm	Start End	Miss	Ions	Peptide
1088.5316	1087.5243	1087.5702	-42.19	**388- 396**	**0**	**---**	R.NFAIGYYLK.E
1105.5586	1104.5513	1104.5927	-37.50	308- 317	1	---	R.YVGKEPSGLR.F
1117.5779	1116.5706	1116.6179	-42.33	246- 255	0	---	K.VADYIPQLAK.F
1304.5841	1303.5768	1303.6271	-38.56	355- 364	0	---	K.FDYVMQFLNK.M
1575.7985	1574.7912	1574.8313	-25.45	280- 292	0	---	K.VPFCLQSCVKPLK.Y
1632.7829	1631.7756	1631.7943	-11.47	383- 396	1	---	R.ESGDRNFAIGYYLK.E
1648.8546	1647.8473	1647.8767	-17.80	203- 216	0	---	K.CVQSNIVLLTQAFR.R
1720.8507	1719.8434	1719.8580	-8.49	293- 307	0	---	K.YAIAVNDLGTEYVHR.Y
1804.9655	1803.9582	1803.9778	-10.83	203- 217	1	---	K.CVQSNIVLLTQAFRR.K
1920.9215	1919.9142	1919.9854	-37.05	86- 106	0	---	K.GSTHPQPGVSPPAAPAAPGPK.D
1922.8947	1921.8874	1921.8993	-6.18	256- 272	0	---	K.FSPDLWGVSVCTVDGQR.H
2007.8592	2006.8519	2006.8792	-13.60	365- 382	0	---	K.MAGNEYVGFSNATFQSER.E
2024.9591	2023.9518	2024.0500	-48.51	184- 201	1	---	R.LTLQTTSDGVMLDKDLFK.K
2144.9847	2143.9774	2144.0361	-27.35	347- 364	1	---	K.QGVNNAEKFDYVMQFLNK.M
2302.0969	2301.0896	2301.1610	-31.01	273- 292	1	---	R.HSTGDTKVPFCLQSCVKPLK.Y
2408.0974	2407.0901	2407.0775	5.23	107- 130	1	---	K.DGPGETDAFGNSEGKELVASGENK.I
2649.2184	2648.2111	2648.2290	-6.74	256- 279	1	---	K.FSPDLWGVSVCTVDGQRHSTGDTK.V

No match to: 1131.7571, 1479.7565, 1567.7170, 1763.7793, 1795.8632, 1891.8845, 1923.9209, 1936.8856, 2013.0567, 2074.9705, 2155.1226, 2330.0893, 2384.9964, 2398.9411, 2401.9405, 2461.0789, 2705.0749, 2719.2274

Supplementary Figure S3. **(A)** MALDI-TOF mass spectrum of the trypsin-digested red asterisk band (peptide mass fingerprint with m/z value). **(B)** Mascot search results for protein identification by peptide mass fingerprint against Swiss Prot database. Comparison of observed and predicted tryptic peptides.

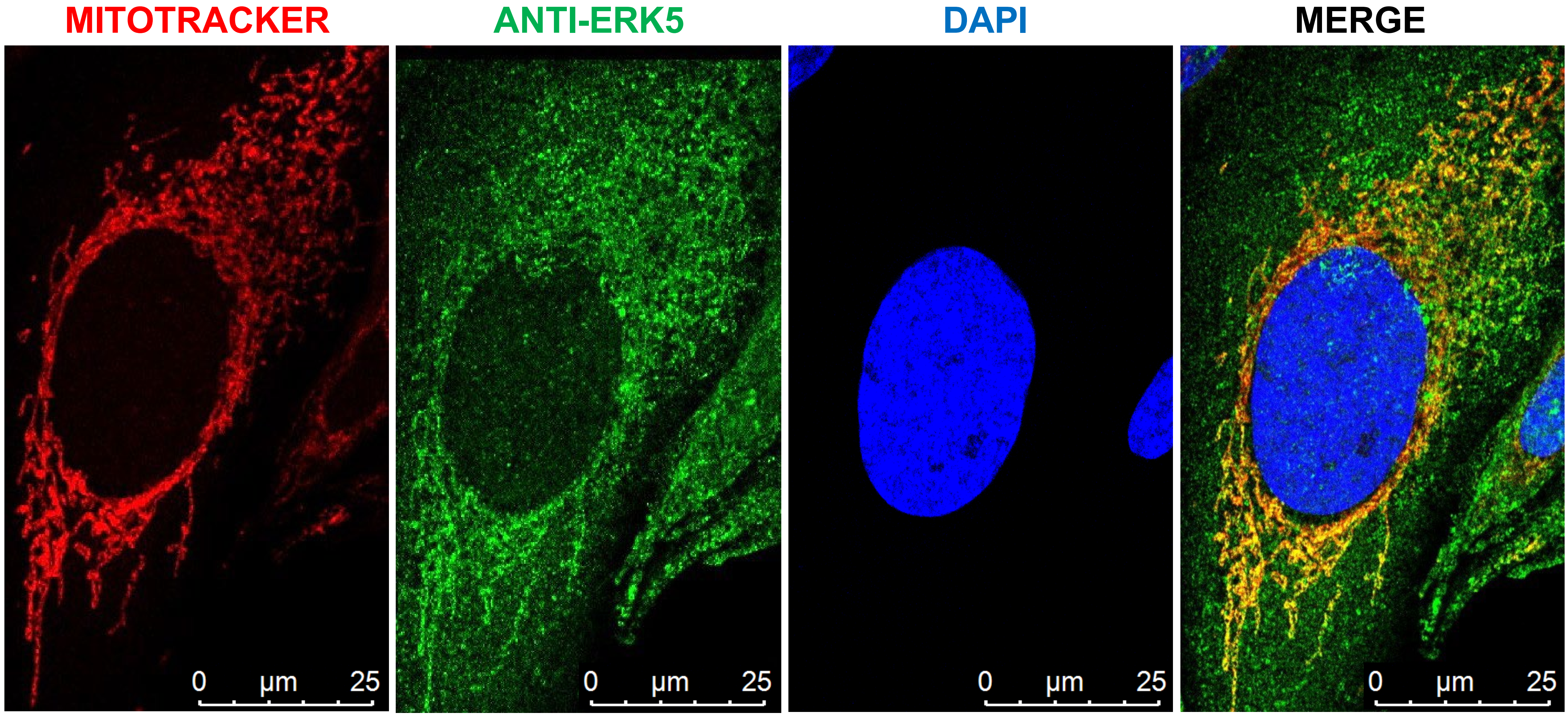

Supplementary Figure S4. Mitochondrial network and ERK5 staining in NP9 cells. Confocal microscopy images showing MitoTracker red staining, anti-ERK5, DAPI and a merged image of all three stains. The yellow signal in the merged image corresponds to coincident mitochondria and ERK5 staining.

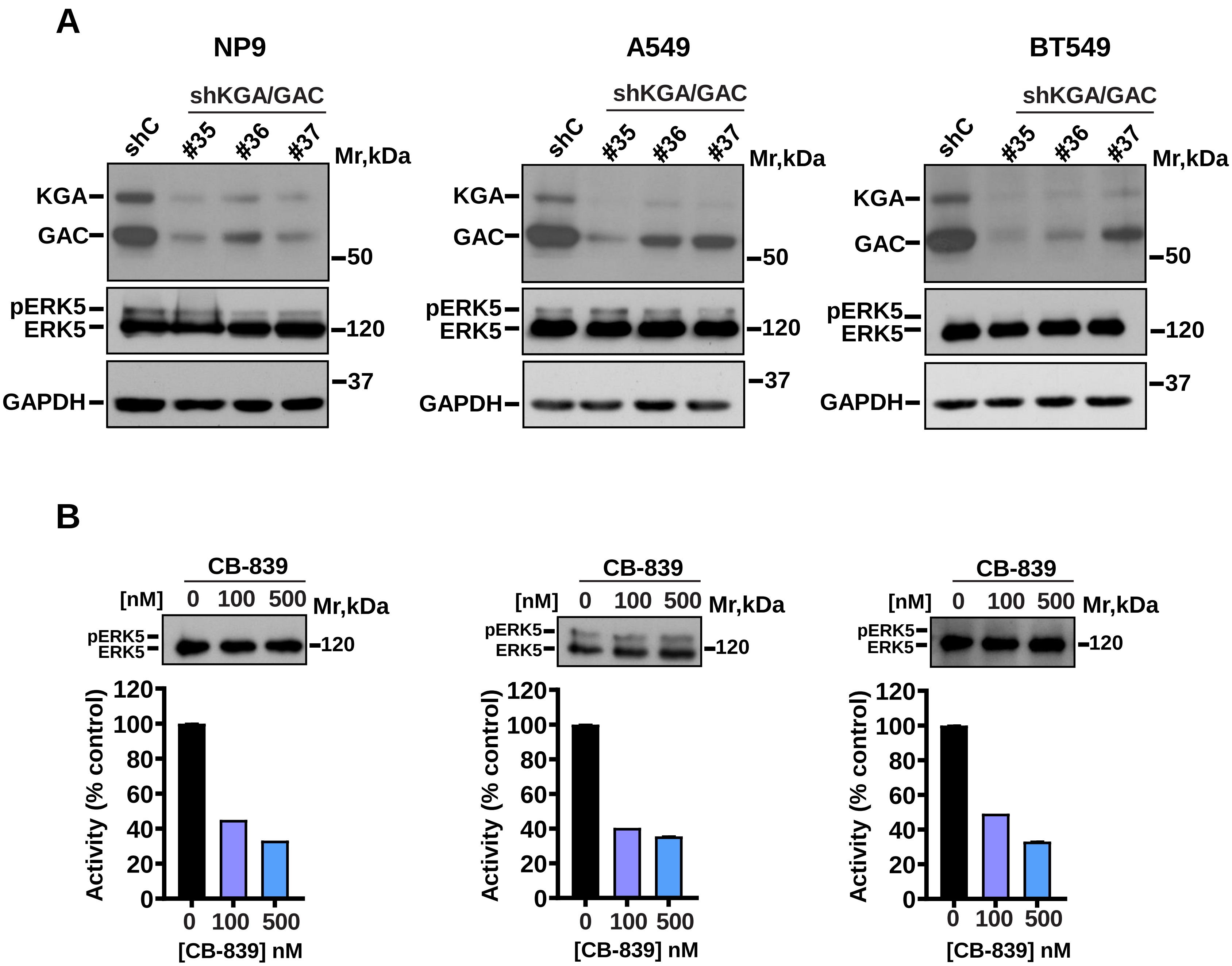

Supplementary Figure S5. Downregulation of KGA/GAC does not affect ERK5. (**A**) KGA/GAC protein expression was downregulated in NP9, A549 and BT549 cell lines with shRNA sequences targeting KGA/GAC (#35, #36 and #37) and a non-coding sequence (shC). 50 µg of protein extracts for NP9 and 100 µg for A549 and BT549, were resolved by SDS-PAGE followed by Western blot using the anti-KGA/GAC antibody (mouse). ERK5 expression was assessed by immunoprecipitation of 1 mg of protein with anti-ERK5 Pro1 antibody and blots were probed with anti-ERK5 C-terminus antibody. GAPDH was used as a loading control. (**B**) Exponentially growing cells were treated with 0.1% DMSO (vehicle) or 100 nM - 500 nM CB-839 for 24 h in culture. ERK5 expression was evaluated in the same way as for panel A. Glutaminase enzymatic activity assays were performed by the OPA method in NP9 cells (left panel), A549 cells (middle panel) and BT549 cells (right panel). Enzyme activity analysis is expressed as percentage respect to control. Bar graphs show mean ± SD of three replicates of an experiment that was repeated twice.

		Peptide Mass Tolerance (ppm)					
		100	50	40	30	20	10
SCORE	GAC	168	174	175	176	114	82
	KGA	141	147	147	148	92	63
COVERAGE (%)	GAC	26	26	26	26	21	18
	KGA	21	21	21	21	16	13

Supplementary Table S1. Mascot search results for protein identification (from the red asterisk band in Figure 1B) by peptide mass fingerprint against Swiss Prot database. The error window for experimental peptide mass values (peptide mass tolerance threshold) was progressively lowered from 100 ppm to 10 ppm. Individual scores higher than 59 were considered significant ($p<0.05$). Percent (%) protein sequence coverage is also indicated.

		Peptide Mass Tolerance (ppm)					
		100	50	40	30	20	10
SCORE	GAC	225	237	190	115	76	41
	KGA	168	178	136	84	60	29
COVERAGE (%)	GAC	39	39	34	26	21	13
	KGA	29	29	25	21	16	9

Supplementary Table S2. Mascot search results for protein identification (from the red asterisk band in Figure S2A) were obtained by comparing the peptide mass fingerprint against the Swiss Prot database. The error window for experimental peptide mass values (peptide mass tolerance threshold) was progressively lowered from 100 ppm to 10 ppm. Scores higher than 59 were considered significant ($p<0.05$). Percent (%) protein sequence coverage is also indicated. NOTE: Although the score values obtained using 10 ppm peptide mass tolerance fall below statistical significance, it is important to report that the only hits detected with the highest score were the two GLS isoforms.
